# Annotations

**Published:** 1902-11-01

**Authors:** 


### Annotations.

### Beneficent Disease.

In addressing the students at University College, Liverpool, Sir Frederick Treves made a point by insisting that we can no longer, after the manner of our forefathers, regard disease as an evil influence distinct from all natural processes and having nothing beneficent in any of its manifestations. The old physicians regarded every symptom of disease as being of necessity wholly noxious and as needing to be stamped out. If the patient vomited, the vomiting must be stopped; if he coughed, the cough must be made to cease; if he failed to take food, he must be made to eat. To the modern physician, however, things appeal in a very different manner. To them there is nothing preternatural about disease. Not only is it the outcome of natural processes, but these processes are themselves, in many cases, marked by a purpose, and that purpose a beneficent one. The time has come when it would rather appear that many of the so-called symptoms of disease are but expressions of a natural effort towards cure, that they are not malign in intent but have for their end the ridding of the body of the very troubles which they are supposed to represent. Take, for example, tuberculosis. Modern pathology teaches that the so-called symptoms of this disease do but represent a valiant attempt on the part of the body to repair an accident, such accident being the entrance of a parasite into the tissues. Take again an inflammation following a septic wound of a finger. The disease, so called, is distressing enough, but the manifestations are no mere outcome of a malign purpose. They are well intended, and have for their object the protection of the body from further parasitic invasion and the elimination of such septic matter as may have been already introduced; and so on. Even the much dreaded peritonitis which to surgeons of the past appeared as the very hand of fate—an impending horror spreading only disaster and death—is now recognised as the operating surgeon's best friend. Times have changed; our views have altered; and we must no longer "fight" disease in the old manner, nor "attack" it with the old weapons.

### Hospital Pneumonia.

The unfortunate death of Mr. Kensit has given rise to a good deal of discussion regarding the temperature of hospital wards. Are patients cured of disease in hospital only to acquire and succumb to another? Is it the bath on entrance—or the daily wash—or the ventilators? Some critics have ventured the opinion that the accuracy of the bed making may have an injurious effect on the patients. It may cause a restless patient some discomfort to be expected to lie absolutely still because the nurse will be angry if he disarranges his blankets, but surely it will not cause him to take pneumonia! It is quite possible that some nurses carry a love of fresh air and cleanliness to excess, forgetting that a patient may get more harm from the fatigue the bath causes him than benefit from the washing of his person, and that doctors, coming in fresh and vigorous from the open air, will notice any closeness in the atmosphere of the ward more quickly than coldness; but it is doubtful if such things occur often, or do harm more than very rarely. And if the bulk of our hospital patients were permitted to enjoy an atmosphere which they regard as "comfortable," the place would soon become in the highest degree insanitary. Moreover, a patient may be brought to hospital suffering obviously from one accident or ailment, but with another disease in an incipient stage. If this develops, must the hospital and hospital discipline be blamed? In the case of Mr. Kensit we have a man who was leading a life of the most intense excitement and strenuous exertion, in which he was inevitably exposed to all variations of temperature. He could hardly escape the risk of chills. An accident caused shock to the system and lowered his vitality, and a chill which in other circumstances might have been shaken off with little or no inconvenience developed into an acute disease, which, unhappily, had a fatal termination. Had Mr. Kensit been taken to a private house and died there, this explanation would probably have been accepted without demur, but because he died in a public institution, and there, not as a direct result of the injury he received, the critics must needs find an excuse for falling foul of our hospitals. Is it fair or reasonable?

### Post-operative Neurasthenia.

We are all accustomed to the condition of neurasthenia which sometimes occurs in those who have received a shake in a railway accident, even though no positive lesion of any organ may be demonstrable. It is a trying enough ailment to the sufferer, and none the less trying from the fact that his enemies and some of his friends are generally ready to tell him quite frankly that he is shamming, and that if he would but stir himself a bit he would be right again directly. Of course he would not be anything of the sort, the malady being far too deeply rooted for any such immediate recovery to be expected. This, however, is not the point to which we would here refer, but rather the fact which we believe is far from being fully recognised, that a condition of the same nature may follow surgical operations and may sadly interfere with complete recovery, notwithstanding the brilliancy with which the operative part of the business has been performed. Professor Clifford Allbutt has recently drawn attention to this subject. He says that he has had many occasions of observing a certain ill-consequence of surgical operations—operations not always severe—which he is disposed to call post-operative neurasthenia. The operation being over and the wound healed, the surgeon assures his patient, to the best of his belief of course, that although he may be in need of a holiday he is practically cured. The surgeon does not see, says Professor Allbutt, what the family physician sees; that, so far from this being the case, the patient is anything but himself again. Week after week he drags along, doing his duty with labour and reluctance, avoiding all engagements he can shirk, and soon knocked up by no excessive demands of work or pleasure. Whether this consequence be attributable to the anæsthetic, "to the unfelt attack upon his inwards," or to the mental stress of the whole affair, it is hard to say, but it is a condition to beware of.

